# Melatonin Alleviates Renal Injury in Mouse Model of Sepsis

**DOI:** 10.3389/fphar.2021.697643

**Published:** 2021-09-02

**Authors:** Liyang Chen, Zhijian Han, Zhiguang Shi, Chao Liu, Qiulun Lu

**Affiliations:** ^1^ Key Laboratory of Cardiovascular and Cerebrovascular Medicine, School of Pharmacy, Nanjing Medical University, Nanjing, China; ^2^ The Department of Urology, The First Affiliated Hospital of Nanjing Medical University, Nanjing Medical University, Nanjing, China; ^3^ Hubei Key Laboratory of Diabetes and Angiopathy, Hubei University of Science and Technology, Xianning, China

**Keywords:** melatonin, sepsis, ROS (reactive oxygen species), acute kidney injury, renal dysfunction

## Abstract

Melatonin (N-acetyl-5-methoxytryptamine; MLT) has been shown to have a renal-protective effect against kidney injury. However, the mechanisms underlying the protective role of MLT in sepsis-induced renal injury are yet to be revealed. In this study, MLT alleviated renal dysfunction with the increase of BUN (blood urea nitrogen) and SCR (serum creatinine) and reduction of fibrosis in the CLP (cecal ligation puncture) model. RNA-seq analysis showed that MLT repressed the oxidant stress in response to kidney injury. Our *in vitro* study showed that MLT suppresses LPS-induced accumulation of ROS (reactive oxygen species) production *via* SOD2 downregulation and Nox4 upregulation in HK-2 cells. Furthermore, we found that MLT alleviated the inflammatory response, with the mRNA-level reduction of *Il-1α*, *Il-1β, Mcp-1*, and *Tgf-β1*. Taken together, in evaluating the therapeutic effect of MLT on sepsis-induced acute kidney injury, the results showed that MLT alleviated renal damage by regulating the production of ROS.

## Introduction

Sepsis is considered as a complicated chain of systemic reactions in response to the imbalance between the host immune response and the pathogenic microorganism, causing septic shock and multiple organ dysfunction (Gustot, 2011). Acute kidney injury (AKI) is a common complication that accounts for death in patients with sepsis, and the incidence of AKI is increasing year by year. The morbidity of AKI in community hospitals is as low as 2%, whereas in large medical institutions, rates can be more than 20% of all hospitalizations (Bellomo et al., 2012).

AKI is an independent prognostic risk factor for septic patients. Although medical care has significantly improved in recent years, the overall mortality has not decreased. Because of the difficulty of AKI treatment, prevention is especially critical. The previous studies revealed that the prevention of MMP (matrix metalloproteinase) activation may be beneficial for the prevention of kidney injury (Gonsalez et al., 2019). Previous study has shown that the modulation of oxidative stress also affects AKI (Aksu et al., 2011). However, there is still no effective pharmacological intervention to treat or reverse AKI.

There are many factors involved in the pathological process of AKI, such as reactive oxygen species (ROS), endoplasmic reticulum stress (ERS), and epithelial–mesenchymal transition (EMT). Under normal or steady-state conditions, the production of the ROS is maintained in balance (Adler and Huang, 2004). However, under pathological conditions, such as sepsis or other stresses, the broken renal microcirculation causes the disruption of the homeostasis of ROS, resulting in pathogenic phenotypes, such as fibrosis and hypoxemia, which leads to renal dysfunction. Once kidney injury occurs, the accumulation of ROS production will cause the reduction of ATP levels and increased inflammatory mediators. This in turn increases intracellular sodium concentrations and leads to an overload of intracellular and mitochondrial calcium (Rabelink and van Zonneveld, 2006). Due to the devastating effects of ROS on the kidney, a new drug is urgently needed.

Melatonin (MLT), a ubiquitous molecule in nature, is synthesized centrally in the pineal gland of vertebrates (especially mammals) and can be locally synthesized in several types of cells and tissues (Brzezinski, 1997). In past studies, MLT has been shown to have a variety of functions, including anti-inflammatory functions, antitumor functions, and circadian rhythm regulation. MLT has shown a powerful antioxidant effect in various diseases (Maleki Dana et al., 2020). MLT could attenuate tumor growth through its free radical–scavenging ability (Kim et al., 2013). The copper-mediated lipid peroxidation in hepatic homogenates could be reduced by melatonin (Galano et al., 2015). The existence of melatonin could even help to improve the success rate of organ transplantation by reducing the production of ROS (Vairetti et al., 2005). The accumulation of ROS abolished by MLT in response to sepsis remains uncovered.

In this study, we demonstrated that MLT attenuated renal dysfunction in CLP-induced injury in kidney tissues. RNA-seq analysis showed that MLT suppressed the CLP-induced increase for the oxidative response, resulting in declined levels for inflammatory factors. Our work indicated that MLT could be a potential therapeutic strategy for acute kidney injury in sepsis.

## Methods and Materials

### Animals and Experimental Models

C57BL/6 mice (male, aged 8 weeks) were obtained from the Animal Core Facility of Nanjing Medical University. They were housed individually in cages under hygienic conditions and placed at a constant room temperature (23 ± 1°C) and humidity (30–40%) and maintained in a 12-h light/dark cycle room with standard mouse chow and water *ad libitum*. All animal experiments were approved by the Animal Care and Use Committee of Nanjing Medical University and were conducted in accordance with the National Institutes of Health Guide for the Care and Use of Laboratory Animals. Mice subjected to cecal ligation puncture (CLP) treatment were grouped as follows: sham group (*n* = 6), sham + MLT group (*n* = 6), CLP group (*n* = 8), and CLP + MLT group (*n* = 8). Melatonin (MLT, CAS No. 73-31-4, MCE) was dissolved in 0.1% ethanol, and the final melatonin concentration in drinking water was 25 μg/ml. Sham group animals were given drinking water with 0.1% ethanol. The drug was administered for a total of 14 days, and on day 15, the mice were operated upon with cecal ligation puncture.

### Cecal Ligation Puncture

CLP was performed as described previously (Toscano et al., 2011). Briefly, mice were deeply anesthetized with inhaled isoflurane using an anesthetic vaporizer. The cecum was exposed by performing a 1-to-2–cm midline incision on the anterior abdomen; the distal half of the cecum was ligated and punctured once using a 19-G needle in the ligated segment. The cecum was then placed back into the abdomen, 1 ml of sterile saline (pyrogen-free 0.9% NaCl) was administered subcutaneously, and the incision was closed using a suture needle. Kidneys were collected 24 h after the treatment.

### Serum Measurements

Blood was collected from the eyes before termination. After centrifugation of samples, urea nitrogen and creatinine concentrations in blood were determined using commercial kits according to the manufacturer’s instructions.

### Cell Culture and Treatment

The HK-2 cells were maintained in DMEM/F12, consisting of 10% FBS, 100 U/mL penicillin, and 100 U/mL streptomycin. They were maintained in a humidified environment with 5% CO_2_ at 37°C. The HK-2 cells were cultured with MLT (5 μΜ), and 48 h later, cells were incubated with lipopolysaccharide (LPS, L4391, Sigma) at a concentration of 1 μg/ml for 24 h.

### Dihydroethidium Staining

To assess the oxidative stress levels in the samples, the sections of the frozen kidney tissues from mice were stained with dihydroethidium (S0063, Beyotime, China) for 30 min. A fluorescence microscope was used to observe and obtain images. Fluorescence intensity of each group was calculated using ImageJ to analyze the ROS levels.

### Histology Staining

Kidney samples from mice were fixed in 10% phosphate-buffered formalin for 48 h at 4°C, embedded in paraffin. The samples were sliced into 4-μm-thick sections. These sections were stained with hematoxylin–eosin (G1005, Servicebio, China) and Masson trichrome staining (G1006, Servicebio, China). Slices were then observed under a light microscope. Renal interstitial fibrosis was estimated using ImageJ.

### RNA Isolation and Real-Time Quantitative PCR

For tissues and cells, total RNA was extracted as previously described, using Trizol reagent (R401-01, Vazyme, China), and then reverse-transcribed into cDNA using RT SuperMix (R323-01, Vazyme, China), following the manufacturer’s protocol. Quantitative real-time PCR assays were performed using QuantStudio 5 (Appliedbiosystems, Thermo Fisher Scientific), using SYBR Green Mix (Q131-02, Vazyme, China). Gene expression was normalized to 18S or GAPDH mRNA. Experiments were performed in triplicate.

### Western Blot Analysis

Lysates were collected in RIPA buffer (P0013B, Beyotime, China), and total protein was quantified using BCA assay (20201ES76, Yeasen, China). Extracted proteins were then mixed with loading buffer, boiled for 10 min, separated by gel electrophoresis, transferred to a nitrocellulose membrane (EMD Millipore, Darmstadt, Germany), and blotted with a primary antibody and an appropriate secondary antibody. Primary antibodies and dilutions used were as follows: Nox4 (1:1,000; 14347-1-AP, Proteintech), Sod2 (1:1,000; sc-137254, Santa Cruz Biotech), and Tubulin (1:1,000; 66031-1-Ig, Proteintech).

### RNA Sequencing and KEGG Analysis

Samples were collected and flash-frozen in liquid nitrogen and then treated with Trizol at −80°C until RNA extraction. Total RNA was extracted from tissues with Trizol. 1.5% agarose gels were used to monitor the RNA degradation and contamination. RNA purity was checked using a NanoPhotometer® spectrophotometer (IMPLEN, CA, United States). The next RNA concentration was measured using a Qubit® RNA Assay Kit in a Qubit® 3.0 Flurometer (Life Technologies, CA, United States). RNA integrity was assessed using an RNA Nano 6000 Assay Kit of the Agilent Bioanalyzer 2,100 system (Agilent Technologies, CA, United States). These processes were operated by Wuhan Frasergen Genomic Medicine Co., Ltd. Raw reads were processed using trim-galore, and clean reads were mapped to mm 10 from UCSC genome1 using Hisat2 (v2.1.0) with default parameters. Differential analysis was performed using edgeR algorithms with fold change ≥1.5 and *p*-value ≤ 0.01 as the thresholds. KEGG (Kyoto Encyclopedia of Genes and Genomes) pathway enrichment analysis was performed using clusterProfiler to explore the biological processes of differentially expressed genes.

### Statistics

Statistical analyses were performed using Prism 8 (GraphPad) software. All data were expressed as means ± SD. Two-group comparisons were analyzed using a Student’s *t*-test or the nonparametric Wilcoxon rank test whenever appropriate. *p* < 0.05 was considered significant.

## Results

### Melatonin Alleviated Renal Dysfunction in Sepsis-Induced Acute Kidney Injury

**FIGURE 1:**
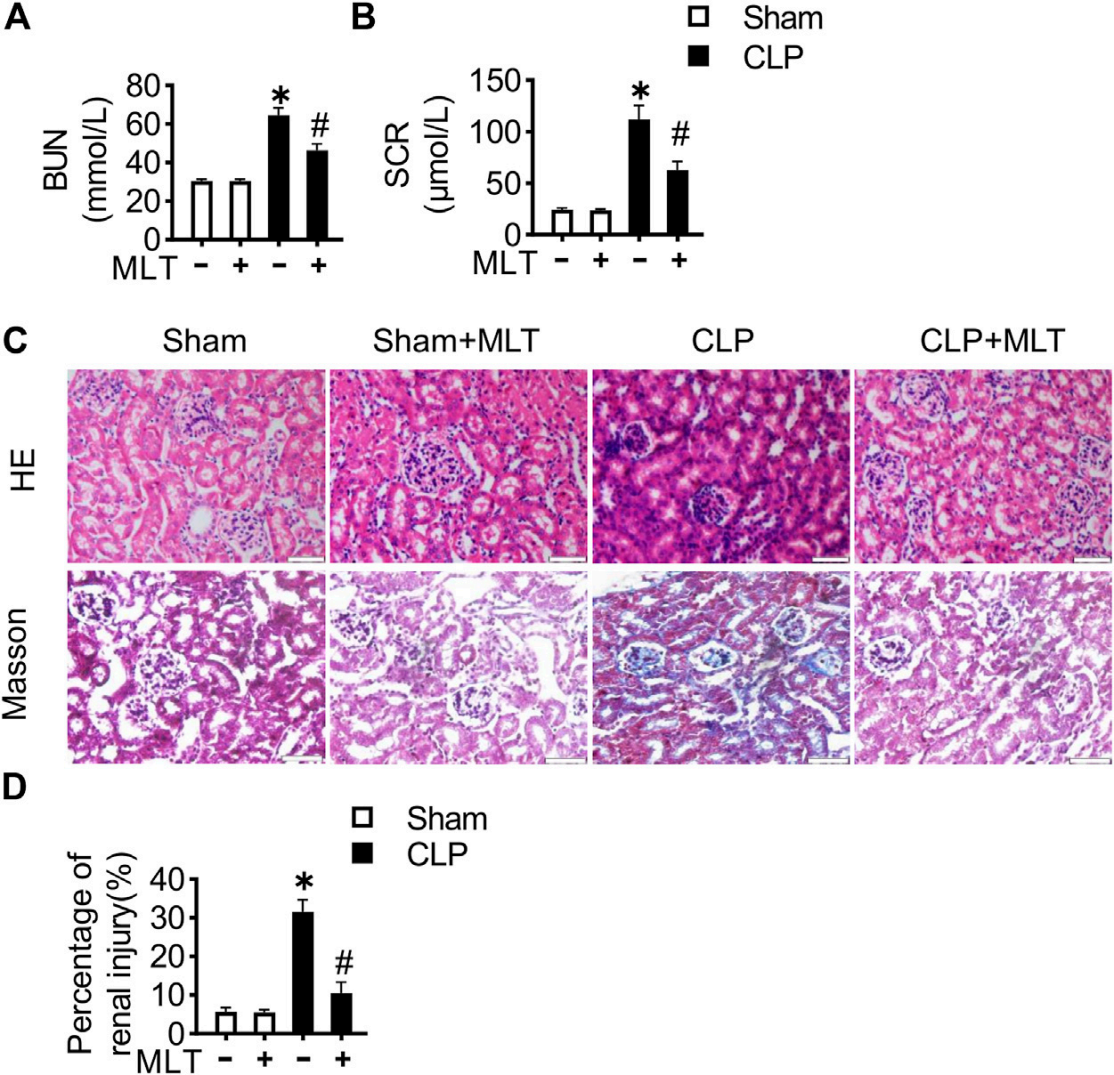
Melatonin alleviates renal dysfunction in sepsis-induced acute kidney injury. 8-week-old mice were treated with melatonin (25 μg/ml) dissolved in drinking water for 14 days. Effects of MLT on the concentration of serum **(A)** BUN and **(B)** SCR (* vs. sham, *p* < 0.05; # vs. CLP, *p* < 0.05). **(C)** Representative images of HE staining and Masson trichrome staining of kidney tissues (scale bar = 50 µm). **(D)** Percentage of renal injury was quantified (* vs. sham, *p* < 0.05; # vs. CLP, *p* < 0.05).

We detected the therapeutic effects of MLT for renal function during the process of AKI in the CLP mouse model, which is the golden and world-wide accepted mouse model for sepsis. Consistent with previous reports, the mice with CLP surgery showed increases in BUN and SCR, compared with the sham group (Figures 1A,B). Furthermore, the intervention of melatonin significantly improved renal function, marked by reduction for BUN and SCR. HE (hematoxylin–eosin) staining with the sections from kidney tissues showed that the CLP group exhibited significant levels of inflammatory cell infiltration, and the renal tubules were swelling (Figure 1C). The renal fibrosis was detected with Masson trichrome staining, showing that the injury was severe in both renal tubules and glomeruli of the CLP group (Figure 1D). Melatonin obviously suppressed the CLP-caused increase for cell infiltration and the renal fibrosis. These results indicated that melatonin played a beneficial role in mediating renal function in the mouse model of CLP.

### Melatonin Relieved Inflammation in CLP-Induced Mice

**FIGURE 2:**
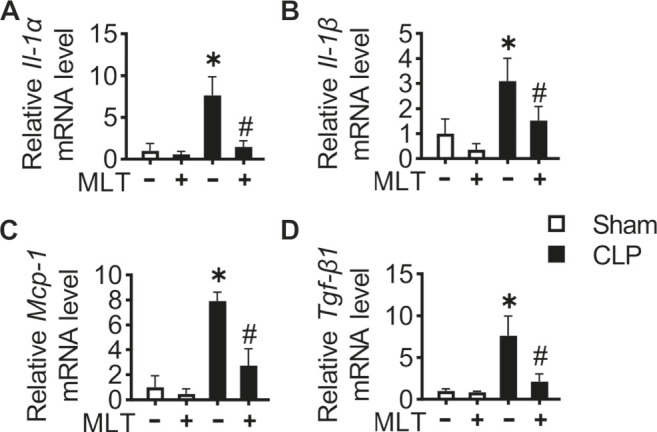
Melatonin relieves inflammation in CLP-induced mice. Kidney tissue inflammatory cytokine *Il-1α* **(A)**, *Il-1β* **(B)**, *Mcp-1* **(C)**, and *Tgf-β1* **(D)** mRNA levels are measured using RT-PCR (* vs. sham, *p* < 0.05; # vs. CLP, *p* < 0.05).

To further confirm the protective role of melatonin in AKI, the pre-inflammatory factors were studied. Real-time PCR assays were performed with the kidney tissues from mice, showing that the mRNA levels for *Il-1α*, *Il-1β*, *Mcp-1*, and *Tgf-β1* were increased in the CLP group compared with the sham (Figures 2A–D). The increased levels of such pro-inflammatory factors were significantly suppressed after melatonin treatment. Taken together, these results showed the anti-inflammatory role of melatonin, further verifying the renal-protective role for melatonin in CLP-caused AKI.

### Melatonin Reduced ROS in CLP-Induced Mice

**FIGURE 3:**
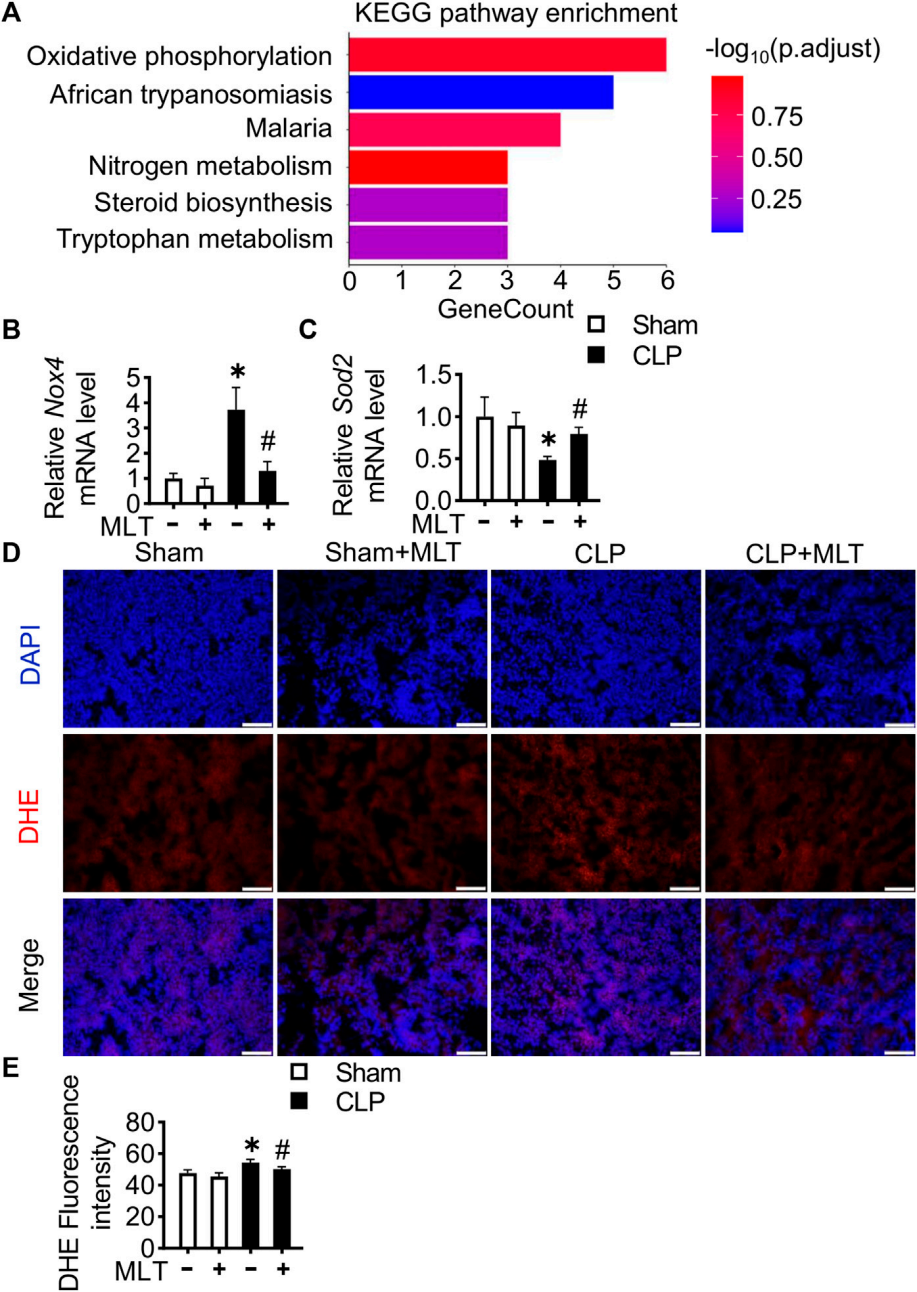
Melatonin reduces ROS in CLP-induced mice. **(A)** KEGG annotation for the differentially expressed genes between the CLP group and the CLP + MLT group. Kidney tissue *Nox4* **(B)** and *Sod2* **(C)** mRNA levels are measured using RT-PCR (* vs. sham, *p* < 0.05; # vs. CLP, *p* < 0.05). **(D)** Representative images of DHE staining of kidney tissues (scale bar = 200 µm). **(E)** ROS production was quantified and presented as the mean fluorescence intensity (* vs. sham, *p* < 0.05; # vs. CLP, *p* < 0.05).

To elucidate the mechanism underlying how melatonin performed a protective role in AKI, RNA-sequencing analysis was performed using kidney tissues from mice. KEGG (Kyoto Encyclopedia of Genes and Genomes) analysis showed that oxidant stress was obviously repressed after melatonin treatment in mice suffering with CLP (Figure 3A). To further confirm the high-throughput RNA-seq result, real-time PCR assays were performed, showing that melatonin repressed the CLP-induced increase for the mRNA level of a typical oxidative factor, *Nox4* (Figure 3B), which is the major subtype for the Nox family in the kidney. Meanwhile, melatonin abolished the reduction of *Sod2* mRNA levels caused by kidney injury (Figure 3C). Additionally, a DHE fluorescent probe was used to detect ROS levels in renal tissues from mice from different groups. Compared with the CLP group, the level of ROS in the kidney was reduced after melatonin treatment (Figures 3D,E). These results indicated that melatonin could reduce renal injury by inhibiting the production of ROS.

### Melatonin Relieved Inflammation in HK-2 Cells Treated With LPS

**FIGURE 4:**
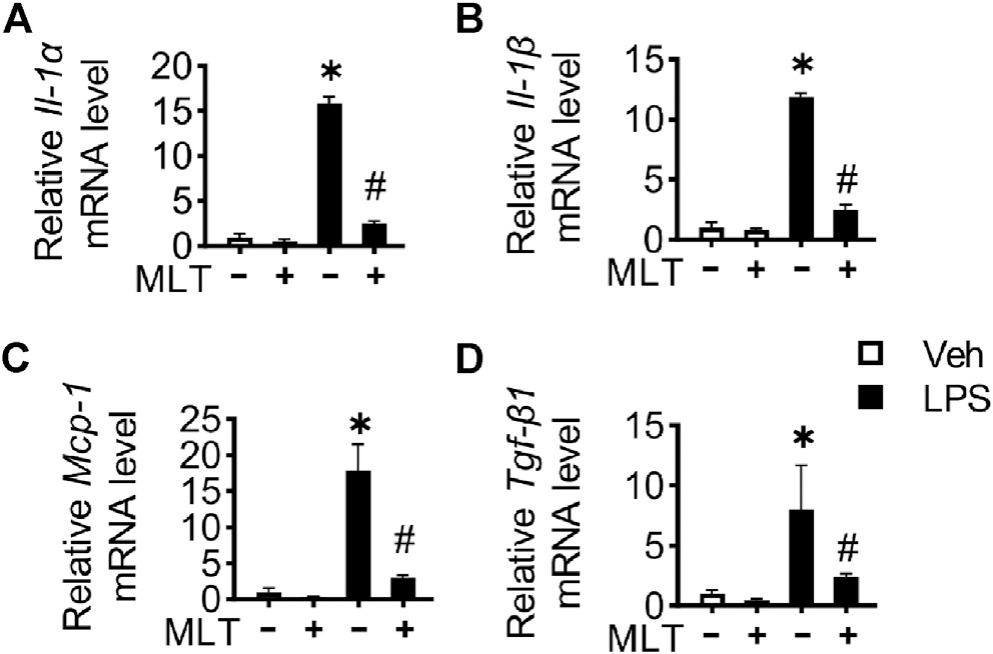
Melatonin relieves inflammation in HK-2 cells treated with LPS. HK-2 cell inflammatory cytokine *Il-1α* **(A)**, *Il-1β* **(B)**, *Mcp-1* **(C)**, and *Tgf-β1* **(D)** mRNA levels are measured using RT-PCR (* vs. Veh, *p* < 0.05; # vs. LPS, *p* < 0.05).

The renal protective role of melatonin was confirmed *in vitro*. The mRNA levels of *Il-1α*, *Il-1β*, *Mcp-1*, and *Tgf-β1* in LPS-induced cells were significantly increased compared to that of the vehicle, while melatonin pretreatment significantly reduced the level of inflammatory cytokines induced by LPS (Figures 4A–D).

### Melatonin Reduced ROS Production in LPS-Treated HK-2 Cells

**FIGURE 5:**
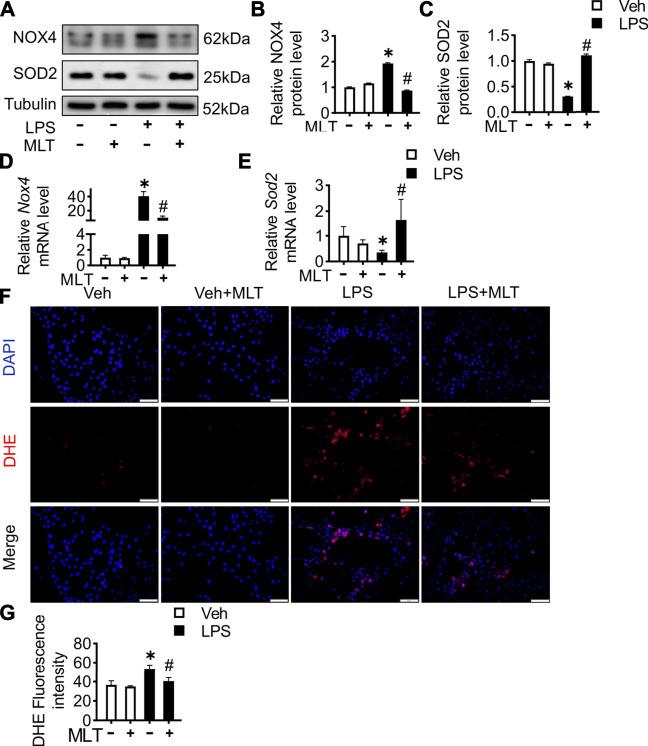
Melatonin reduces ROS production in LPS-treated HK-2 cells. The HK-2 cells were cultured with MLT (5 μM), and 48 h later, cells was incubated with lipopolysaccharide (LPS, L4391, Sigma) at a concentration of 1 μg/ml for 24 h. **(A)** Protein expression levels of *NOX4* and *SOD2* in the HK-2 cells were measured by Western blotting. The expression levels of NOX4 **(B)** and SOD2 **(C)** were quantified (* vs. Veh, *p* < 0.05; # vs. LPS, *p* < 0.05). *Nox4* **(D)** and *Sod2* **(E)** mRNA levels of cells are measured using RT-PCR (* vs. Veh, *p* < 0.05; # vs. LPS, *p* < 0.05). **(F)** Representative images of DHE staining of HK-2 cells (scale bar = 50 µm). **(G)** ROS production was quantified and presented as the mean fluorescence intensity (* vs. Veh, *p* < 0.05; # vs. LPS, *p* < 0.05).

To illustrate the renal protective effect of melatonin, *in vitro* experiments using HK-2 cells were performed. The protein expression level of *Nox4* was increased, while SOD2 was reduced in the LPS group (Figures 5A–C). Similar to the prior mice study, melatonin also played an antioxidative role in HK-2 cells. The same protective function could also be found with the expression of *Nox4* and *Sod2* mRNA (Figures 5D,E). To evaluate the ROS level in the cells, a DHE fluorescent probe was used. The results revealed that melatonin could abolish the accumulation of ROS in LPS-treated HK-2 cells (Figures 5F,G).

## Discussion

This study found that melatonin can play a protective role in AKI caused by sepsis. Mechanistically, melatonin alleviated the inflammatory response and fibrosis induced by sepsis by scavenging ROS. Our results indicate that melatonin is a potential therapeutic strategy for sepsis-caused AKI in the clinic.

**FIGURE 6:**
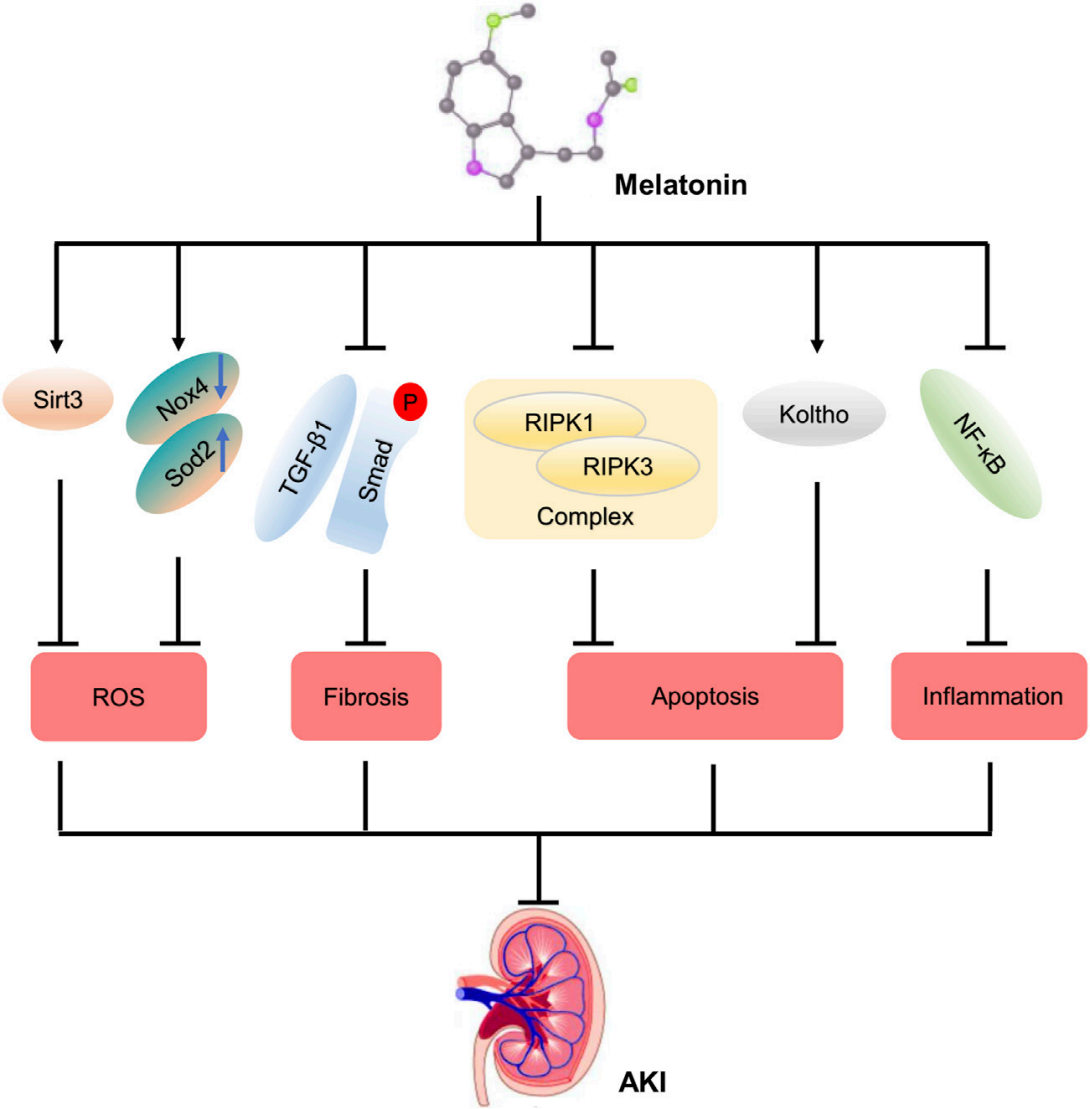
Potential protective mechanism of melatonin on AKI. A diagrammatic representation of some of the protective mechanism of melatonin on AKI. Abbreviations: Sirt3, Sirtuin-3; TGF-β1, transforming growth factor-β1; RIPK, receptor-interacting protein kinase; NF-κB, nuclear factor kappa-B; ROS, reactive oxygen species; AKI, acute kidney injury.

MLT can alleviate renal dysfunction and even failure. Numerous studies have showed that MLT attenuates acute kidney injury *via* different pathways (Figure 6). MLT protects kidney function *via* the Sirt3-mediated oxidant response in contrast-caused AKI (Zhang et al., 2021). MLT alleviates cellular apoptosis after kidney injury caused by cisplatin, inhibiting the NF-κB–mediated inflammatory response and RIPK1/RIPK3 complex–mediated necroptosis (Kim et al., 2019). Meanwhile, MLT increases the expression of an anti-aging protein, Koltho, and prevents cellular apoptosis, resulting in maintaining the renal function (Ko et al., 2019). Additionally, the process of renal fibrosis in AKI is attenuated by MLT *via* inactivation of the TGFβ pathway (Chen et al., 2019). Herein, we identified that MLT was a scavenger of ROS to alleviate renal dysfunction.

Previous studies have used pharmacological interventions to target the accumulation of ROS production in AKI. However, our studies identified that melatonin helped to maintain renal function in sepsis-induced AKI. MLT has been demonstrated to exert antioxidation effects in the skin, the intestine, and the lungs, and it can also assist in relieving chronic pelvic pain in women with endometriosis (Maleki Dana et al., 2020). It has been shown that MLT can decrease the kidney damage induced by insulin resistance (IR), unilateral ureteral occlusion (UUO), and severe burns by acting as an antioxidation and antiapoptotic agent (Promsan and Lungkaphin, 2020). In this study, an AKI mouse model was established *via* CLP surgery with the phenotype of renal tissue damage. The results revealed that the extent of tissue damage in the CLP group was the highest, whereas the extent of tissue damage was reduced by melatonin.

Our studies revealed that the anti-inflammatory function of melatonin was realized through impairing the accumulation of ROS production. Melatonin has a similar effect in other diseases. Mitochondrial dysfunction is both the cause and consequence of excessive free radical formation and inflammatory responses. In Alzheimer’s disease models, changes in proinflammatory cytokines and antioxidative factors caused by melatonin have been only occasionally observed (Calabrese et al., 2020). Melatonin has been proved to protect mitochondrial integrity and function *via* diminished production of ROS, which is inhibited by reduced inflammatory factors (Hardeland et al., 2015). Melatonin has effects against a pro-inflammatory and pro-oxidant status in the lung of old SAMP8 mice (Puig et al., 2016). Melatonin is also found to alleviate CKD as an anti-inflammatory and antioxidative treatment by ameliorating sleep disorders (Maung et al., 2016). Our studies revealed that the renal protective function of melatonin was realized through inhibiting the production of ROS.

However, it was reported that melatonin has a reno-protective role to prevent sepsis-induced renal injury (Dai et al., 2019). In their study, melatonin was administrated 14 days before surgery, identifying the preventability of melatonin in acute kidney injury. Our study was focusing on the therapeutic role of melatonin in sepsis-induced renal injury. What is more, the mechanism underlying how MLT alleviates renal dysfunction in response to sepsis was unbiasedly uncovered by RNA sequencing, identifying the redox signaling pathway that occurred in MLT-controlled renal function. We also verified the molecular mechanism both *in vivo* and *in vitro*, making the results more convincing and logical. Last, MLT was dissolved in drinking water in our study, which is closer to the use in clinics.

In summary, we found that melatonin can protect and maintain the renal function in CLP-induced AKI. Melatonin alleviates renal fibrosis and inflammatory response *via* reduction of ROS production. In clinics, melatonin is a potential protective strategy for sepsis-induced AKI.

## Data Availability

The raw data supporting the conclusions of this article will be made available by the authors, without undue reservation. The data for RNA-seq presented in the study are deposited in the Gene Expression Omnibus and the accession number is GSE178780.
